# Genome-Based Genetic Tool Development for *Bacillus methanolicus*: Theta- and Rolling Circle-Replicating Plasmids for Inducible Gene Expression and Application to Methanol-Based Cadaverine Production

**DOI:** 10.3389/fmicb.2016.01481

**Published:** 2016-09-22

**Authors:** Marta Irla, Tonje M. B. Heggeset, Ingemar Nærdal, Lidia Paul, Tone Haugen, Simone B. Le, Trygve Brautaset, Volker F. Wendisch

**Affiliations:** ^1^Genetics of Prokaryotes, Faculty of Biology and CeBiTec, Bielefeld University Bielefeld, Germany; ^2^SINTEF Materials and Chemistry, Department of Biotechnology and Nanomedicine Trondheim, Norway; ^3^Department of Biotechnology, Norwegian University of Science and Technology Trondheim, Norway

**Keywords:** *Bacillus methanolicus*, thermophile, methylotroph, genetic tool box, theta-replicating plasmids, gene expression

## Abstract

*Bacillus methanolicus* is a thermophilic methylotroph able to overproduce amino acids from methanol, a substrate not used for human or animal nutrition. Based on our previous RNA-seq analysis a mannitol inducible promoter and a putative mannitol activator gene *mtlR* were identified. The mannitol inducible promoter was applied for controlled gene expression using fluorescent reporter proteins and a flow cytometry analysis, and improved by changing the -35 promoter region and by co-expression of the *mtlR* regulator gene. For independent complementary gene expression control, the heterologous xylose-inducible system from *B. megaterium* was employed and a two-plasmid gene expression system was developed. Four different replicons for expression vectors were compared with respect to their copy number and stability. As an application example, methanol-based production of cadaverine was shown to be improved from 6.5 to 10.2 g/L when a heterologous lysine decarboxylase gene *cadA* was expressed from a theta-replicating rather than a rolling-circle replicating vector. The current work on inducible promoter systems and compatible theta- or rolling circle-replicating vectors is an important extension of the poorly developed *B. methanolicus* genetic toolbox, valuable for genetic engineering and further exploration of this bacterium.

## Introduction

*Bacillus methanolicus* is a thermophilic bacterium, able to grow on methanol as a sole carbon and energy source (Schendel et al., 1990; Arfman et al., 1992). The growth of *B. methanolicus* occurs in a wide temperature range between 37 and 60°C, with an optimum at 50°C. It was, however, observed that a rapid change of growth temperature from 50 to 37°C leads to the initiation of sporulation processes in the wild type strain MGA3, specifically the upregulation of stage VI sporulation protein D, the anti-sigma F factor antagonist SpoIIAA, and the stage IV sporulation protein A and the downregulation of two proteins which belong to the flagellar apparatus (Schendel et al., 1990; Müller et al., 2014). *B. methanolicus* MGA3 produces 60 g/L of L-glutamate in methanol-controlled high cell density fed-batch fermentations (Schendel et al., 2000; Heggeset et al., 2012). Currently, *Corynebacterium glutamicum* is typically used for the industrial production of L-glutamate in fermentative processes with the most common carbon sources being molasses and sugar cane, and the global annual consumption reaching 3.2 million tons (IHS Chemical, 2016). The second largest product of the amino acid market is L-lysine, a feed additive with an annual demand exceeding 2 million tons (Zahoor et al., 2012). *B. methanolicus* does not naturally overproduce this amino acid, however, during the last two decades several strategies have been employed to generate L-lysine producing strains. To date, the classical mutants of *B. methanolicus* produce up to 65 g/L of L-lysine in high cell density methanol fed-batch fermentations (Brautaset et al., 2010). Furthermore, it was shown both in wild type and lysine producing strains that heterologous expression of a lysine decarboxylase enables the synthesis of cadaverine (Nærdal et al., 2015). Cadaverine, also known as 1,5-diaminopentane, is a five-carbon linear aliphatic diamine (Schneider and Wendisch, 2011; Shimizu, 2013), that finds applications in the (bio)plastics industry since polycondensation of cadaverine with dicarboxylic acids yields polyamides or nylons of the AA,BB-type (Shimizu et al., 2003; Wendisch, 2014). The most significant advantage of *B. methanolicus* for the use in the amino acid industry is its ability to utilize methanol as a carbon source in combination with a high growth temperature, which leads to a reduced need for cooling. Methanol is a cheap, non-food alternative to raw materials commonly used in the biotechnological processes (Müller et al., 2015a). In the recent years, considerable progress has been made in the elucidation of the methanol utilization pathway starting from sequencing of the full genome (Heggeset et al., 2012; Irla et al., 2014), characterization of the enzymes involved in the methanol oxidation and the ribulose monophosphate (RuMP) pathway (Krog et al., 2013; Stolzenberger et al., 2013a,b; Markert et al., 2014; Ochsner et al., 2014; Wu et al., 2016), unraveling of the transcriptome by the means of microarray analysis (Heggeset et al., 2012) and RNA-seq (Irla et al., 2015), of the proteome (Müller et al., 2014), and the metabolome (Kiefer et al., 2015; Müller et al., 2015b). These findings enabled a better understanding of the metabolic processes taking place during growth on methanol, but also on the limited number of alternative C-sources for this facultative methylotroph, in particular on mannitol.

The obvious suitability of *B. methanolicus* for industrial application has been the main motivation behind the extensive work on the development of metabolic engineering tools. The first attempts included random mutagenesis approaches towards increased L-lysine production (Hanson et al., 1996; Brautaset et al., 2010). Furthermore, the protocol for the protoplast transformation with plasmid DNA was developed and several different origins of replication were tested for their transformation efficiency and stability (Cue et al., 1997). The protoplast-based transformation protocols are known to be laborious and difficult to perform, for this reason a more versatile electroporation procedure was developed, and for the first time the *mdh* promoter (*mp*) was used to establish plasmid based gene expression (Jakobsen et al., 2006). The only alternative vector that has been used for the heterologous gene expression thus far is pNW33N with a *gfp* gene cloned under control of the *mdh* promoter (Nilasari et al., 2012).

Despite the fact that some progress has been made in genetic manipulation of *B. methanolicus*, and that L-lysine and cadaverine producing strains have been created by the plasmid-based gene expression, the available toolbox is a limiting factor for the development of industrially relevant *B. methanolicus* strains. Here, we present the expansion of the metabolic engineering tool box by the addition of two new expression vectors and the establishment and development of xylose- and mannitol-inducible promoter systems.

## Materials and Methods

### Strains, Plasmids, and Primers

All strains, plasmids, and primers constructed and used in this study are listed in the Supplementary Tables. *B. methanolicus* MGA3 was used as the expression host, *Escherichia coli* strain DH5α (Stratagene) was used as the general cloning host.

### Molecular Cloning

All standard recombinant DNA procedures were performed as described by Sambrook and Russell (2001). Plasmid DNA was introduced into chemically competent *E. coli* cells (Higa and Mandel, 1970; Hanahan, 1983). Total DNA was isolated from *B. methanolicus* using the MasterPure^TM^ Gram Positive DNA Purification Kit (Epicenter) or as previously described (Eikmanns et al., 1994). The NucleoSpin^®^ Gel and PCR Clean-up kit (Machery-Nagel) and the Qiaquick PCR Purification and Gel Extraction kits (Qiagen) were used for PCR purification and gel extraction. Plasmids were isolated using the GeneJET Plasmid Miniprep Kit (Thermo Fisher Scientific) or the Wizard^®^ Plus SV Minipreps (Promega). Plasmid backbones were amplified with PfuTurbo DNA polymerase (Agilent), inserts with ALLin^TM^ HiFi DNA Polymerase (highQ) or the Expand^TM^ High Fidelity PCR System (Roche). Dephosphorylation of plasmid DNA was performed using Antarctic Phosphatase or Calf Intestinal Alkaline Phosphatase (New England Biolabs). The DNA fragments were joined either with Rapid DNA Ligation Kit (Roche), T4 DNA ligase (New England Biolabs) or by the means the isothermal DNA assembly (Gibson et al., 2009). For colony PCR the Taq polymerase (New England Biolabs) was used. Site-directed mutagenesis was performed essentially as described by Liu and Naismith (2008) using Pfu polymerase (Agilent). All cloned DNA fragments and introduced mutations were verified by sequencing. *B. methanolicus* competent cells were prepared according to Jakobsen et al. (2006). SOBsuc plates [1% (w/v) agar] supplemented with suitable antibiotics were used instead of regeneration plates. SOBsuc medium is SOB medium (Difco) supplemented with 0.25 M sucrose. Electroporation was performed as previously described (Jakobsen et al., 2006).

### Media and Cultivation Conditions

*Escherichia coli* strains were cultivated at 37°C in Lysogeny Broth (LB) or on LB–agar plates supplemented with antibiotics (ampicillin 200 μg/mL, chloramphenicol 30 μg/mL, kanamycin 50 μg/mL) when relevant. Unless otherwise stated, *B. methanolicus* strains were cultured at 50°C in MVcMY minimal medium with 200 mM methanol as previously described (Brautaset et al., 2004). When appropriate, media were supplemented with kanamycin 50 μg/mL (or 10 μg/mL) and/or chloramphenicol 5 μg/mL. Inducers were used at the following concentrations: mannitol [2.5, 5.0, 12.5, 25, 50, and 55 mM (1%)], arabitol (50 mM), ribitol (50 mM), xylitol (50 mM), xylose [0.01, 0.05, 0.1, 0.5, or 1% (w/v)], or CuSO_4_ (10, 20, 50, 100, and 200 μM). All experiments were performed in triplicates.

### β-Galactosidase (LacZ) Activity Assay

For LacZ enzymatic assays, overnight cultures of *B. methanolicus* strains MGA3 (pTH1mp-*lacZ*), MGA3 (pTH1xp-*lacZ*), MGA3 (pTH1cup-*lacZ*), MGA3 (pTH1mtlAp-*lacZ*), or MGA3 (pHP13), were diluted to OD_600_ 0.2 in fresh medium with appropriate antibiotics. When the cultures reached OD_600_ = 0.5, they were split in two equal halves. Inducer (50 μM CuSO_4_, 1% (w/v) xylose, or 1% (w/v) mannitol) was added to one of the two and growth was continued until OD_600_ 1–1.5. Cells were harvested by centrifugation (5000 *g*, 10 min, 4°C) and the pellets were stored at -80°C. Cells were thawed, resuspended in potassium phosphate buffer (100 mM, pH 7.0) (10% of the original volume) and sonicated on ice/water for 10-15 min (Branson Sonifier 250, output control = 3 and duty cycle = 30%). Cellular debris was removed by centrifugation (10000 *g*, 45 min, 4°C) followed by filtration through a 0.2 μm sterile filter. Enzymatic activities were measured by monitoring the liberation of *o*-nitrophenol from *o*-nitrophenyl β-D-galactopyranoside (ONPG) at 410 nm. 100 mM potassium phosphate buffer pH 7.0 (910 μl), 68 mM ONPG (30 μl), and 30 mM MgCl_2_ (30 μl) were mixed and the catalysis started by the addition of cell extract (30 μl). The molar extinction coefficient used for *o*-nitrophenyl at 410 nm, pH 7.0 used for calculation is 3500 M^-1^ cm^-1^ and the light path 1 cm. One unit (U) is defined as the amount of enzyme able to convert 1.0 μmol of ONPG per min.

### Flow Cytometry

For the fluorescent activated cell scanning analysis, overnight cultures were diluted to an initial OD_600_ of 0.15 and cultivated for 6 h at 50°C prior to incubation at 37°C for two hours. Samples were centrifuged at 13,000 *g*, 5 min, 4°C, washed twice with cold phosphate-buffered saline (PBS) and resuspended therein to a final OD_600_ of 0.3. The fluorescence was determined in a flow cytometer (Becton Dickinson) using the Kaluza for Gallios Acquisition Software 1.0. The fluorescence emission signal was collected with a 450/50 BP bandpass filter (FL9) for GFPuv and with a 620/30 BP, bandpass (FL3) for mCherry. The following data analysis was performed using Kaluza Analysis Software 1.3.

### Plasmid Stability

To test for stability of plasmid segregation, overnight cultures were diluted in 50 mL fresh medium with and without relevant antibiotics to an initial OD_600_ of 0.05, grown for 12 h (six generations) and then diluted again into fresh medium to an initial OD_600_ of 0.05. This was repeated over the course of the whole experiment. After 6 h from inoculation, 10 mL of the cultures were aliquoted to 100 mL shaking flasks and incubated for 2 h at 37°C, 200 rpm, after which the flow cytometry analysis was carried out. This procedure was repeated every 24 h (every 12 generations) for a total of 5 days (60 generations). The stability is presented as the ratio of cells fluorescent in absence of antibiotics to the cells fluorescent in presence of antibiotics.

### Estimation of Copy Number by the Means of Droplet Digital PCR

Overnight cultures of MGA3 (pHCMC04), MGA3 (pHP13), MGA3 (pNW33Nkan), or MGA3 (pUB110Smp-*lacZ*) were diluted to 2% in fresh medium (supplemented with 5 μg/ml chloramphenicol or 10 μg/ml kanamycin) and cultivated until the mid-exponential growth phase (OD_600_ 2-4). Cell pellets were harvested from 5 ml cultures by centrifugation and total DNA was extracted using the MasterPure^TM^ Gram Positive DNA Purification Kit (Epicenter), followed by an additional purification step using the Agencourt^®^ AMPure XP system (Beckman Coulter). DNA concentrations were determined on a Qubit^®^ 2.0 Fluorometer using the Qubit^®^ dsDNA BR Assay Kit (ThermoFischer Scientific). Twenty microliter ddPCR reaction mixtures containing EvaGreen Supermix (Bio-Rad), primers (0.2 μM) and gDNA template (8 or 20 pg) were prepared according to the manufacturer’s instructions and used for droplet generation (QX200 droplet generator, Bio-Rad). Forty microliter of sample was manually transferred to a 96-well plate and heat-sealed prior to amplification initiated by enzyme activation at 95°C for 5 min, followed by 40 cycles of amplification (95°C for 30 s, 60°C 1 min) and signal stabilization (4°C 5 min, 90°C 5 min), temperature ramp 2.5°C/s. Following amplification, fluorescence intensity was measured in a QX200 Droplet Reader (Bio-Rad) and the signal data were analyzed with QuantaSoft, Version 1.5.38 (Bio-Rad). Primer sequences are listed in the Supplementary Material.

### High Cell Density Fed-Batch Methanol Fermentation

Fed-batch fermentation was performed at 50°C in UMN1 medium using Applikon 3 L fermenters with an initial volume of 0.75 L medium essentially as previously described (Jakobsen et al., 2009; Brautaset et al., 2010). Kanamycin (50 μg/mL) or chloramphenicol (5 μg/mL) was added to the initial batch growth medium, the pH was maintained at 6.5 by automatic addition of 12.5% (w/v) NH_3_ solution, and the dissolved oxygen level was maintained at 30% saturation by increasing the agitation speed and using enriched air (up to 60% O_2_). The methanol concentration in the fermenter was monitored by online analysis of the headspace gas with a mass spectrometer (Balzers Omnistar GSD 300 02). The headspace gas was transferred from the fermenters to the mass spectrometer in insulated heated (60°C) stainless steel tubing. The methanol concentration in the medium was maintained at a set point of 150 mM by automatic addition of methanol feed solution containing methanol, trace metals and antifoam 204 (Sigma), as previously described (Brautaset et al., 2010). All fermentations were run until the carbon dioxide content of the exhaust gas was close to zero (no cell respiration). Bacterial growth was monitored by measuring OD_600_. Dry cell weight was calculated using a conversion factor of one OD_600_ unit corresponding to 0.24 g dry cell weight per liter (Jakobsen et al., 2009). Due to significant increase in the culture volume throughout the fermentation, the biomass, cadaverine, and amino acid concentrations were corrected for the increase in volume and subsequent dilution. A volume correction factor of 1.8 was used for values presented in **Table 2**. The actual concentrations measured in the bioreactors were therefore accordingly lower as described previously (Jakobsen et al., 2009). Samples for determination of volumetric cadaverine and amino acid yields were collected from early exponential phase and throughout the cultivation (10–47 h).

### Measurement of Cadaverine and Amino Acids

Samples were analyzed by RP-HPLC as described previously by Skjerdal et al. (1996) using pre-column derivatization with *o*-phtaldialdehyde and a buffer containing 0.02 M sodium acetate +2% tetrahydrofuran at pH 5.9.

### Detection of α-Amylase Activity

For the detection of α-amylase activity, overnight cultures were diluted to an initial OD_600_ of 0.15 and cultivated for 6-8 h at 50°C. The cultures were diluted to OD_600_ of 1 and 15 μL of the diluted cultures were placed on the appropriate plates in the form of a drop. The plates were incubated for 12 h at 50°C to allow the cell growth and then placed at 37°C for next 24 h for in order to support activity of the heterologous α-amylase. Ten milliliter of iodine solution were placed on the plate in order to visualize the formation of the halo in the starch.

## Results

### Comparison of Different Replicons for Plasmid-Based Gene Expression in *B. methanolicus* MGA3

Genetic engineering of *B. methanolicus* has until now relied on only two plasmids, pNW33N and pHP13. Therefore, we decided to analyze a range of different replicons with regard to their applicability for gene overexpression in *B. methanolicus* MGA3. We compared four different plasmids that were able to replicate: pTH1mp (derived from pHP13), pUB110Smp, pNW33Nmp, and pBV2mp (derived from pHCMC04). As shown in **Table 1**, we have chosen plasmids differing in the copy number, original host organism and the replication mechanism. All rolling circle (RC) plasmids used belong to the pC194/pUB110 family, which is characterized by similarity in Rep protein and the sequences of sites involved in the replication with pNW33N and pUB110 sharing identical Rep protein sequences. The pUB110 plasmid is reported to be a high copy number plasmid in *B. subtilis*, pNW33N – medium, pHP13 and pBV2mp – low copy number, both of the low copy number plasmids originate from *B. subtilis*.

**Table 1:** Comparison of properties and *gfpUV* expression from methanol-inducible promoter P*_mp_* of plasmids used in the study.

Plasmid	Parental plasmid	Original host organism	Replication model	Copy number in *Bacillus subtilis*	Copy number in *B. methanolicus^#^*	Median fluorescence intensity of GFPuv [a.u.]
pTH1mp-*gfpuv*	pHP13 (pTA1060)^a^	*B. subtilis*	Rolling circle	5-6^a^	5 ± 1^1^	0.9 ± 0.1
pNW33Nmp-*gfpuv*	pNW33N (pC194)^b^	*S. aureus*	Rolling circle	15aaa^e^	19 ± 2^2^	2.5 ± 0.1
pUB110Smp-*gfpuv*	pUB110^c^	*S. aureus*	Rolling circle	30-50^c^	25 ± 1^3^	3.9 ± 0.0
pBV2mp-*gfpuv*	pHCMC04 (pBS72)^d^	*B. subtilis*	Theta-replication	6^∗f^	3 ± 1^4^	0.2 ± 0.0

^a^*Haima et al., 1987;*^b^*Rhee et al., 2007;*^c^*Gryczan et al., 1978*, ^d^*Nguyen et al., 2005;*^e^*te Riele et al., 1986;*^f^*Titok et al., 2003; bbbPlasmid copy number for parental plasmid pC194; ^∗^Plasmid copy number for derivative of parental plasmid pBS72;*^1^*pHP13;*^2^*pNW33Nkan;*^3^*pUB110Smp-*lacZ*;*^4^*pHCMC04. ^#^For comparison, the copy numbers of the native *B. methanolicus* plasmids pBM19 and pBM69 were 3.4 ± 0.7 and 1.3 ± 0.2, respectively. *B. methanolicus* without vector showed a mean GFPuv fluorescence of 0.1 ± 0.0. Mean values and standard deviations of triplicate shake flask cultures are presented*.

Our initial goal was to characterize the copy number, expression levels and stability of the chosen plasmids in *B. methanolicus* MGA3. To analyze the expression levels, *gfpuv* (Chalfie et al., 1994; Crameri et al., 1996) was used as a reporter controlled by the *mdh* promoter from *B. methanolicus* MGA3. Fluorescence intensity was evaluated during growth in methanol minimal medium by flow cytometry. Using the ddPCR, plasmid copy numbers were estimated for the selected plasmids and, in comparison, for the native MGA3 plasmids pBM19 and pBM69 (**Table 1**). The plasmid pUB110Smp-*gfpuv* showed the highest fluorescence levels among the plasmids tested (**Table 1**), followed by pNW33Nmp-*gfpuv*, pTH1mp-*gfpuv*, and pBV2mp-*gfpuv*, respectively, which was in accordance to the plasmid copy number results (**Table 1**).

**FIGURE 1:**
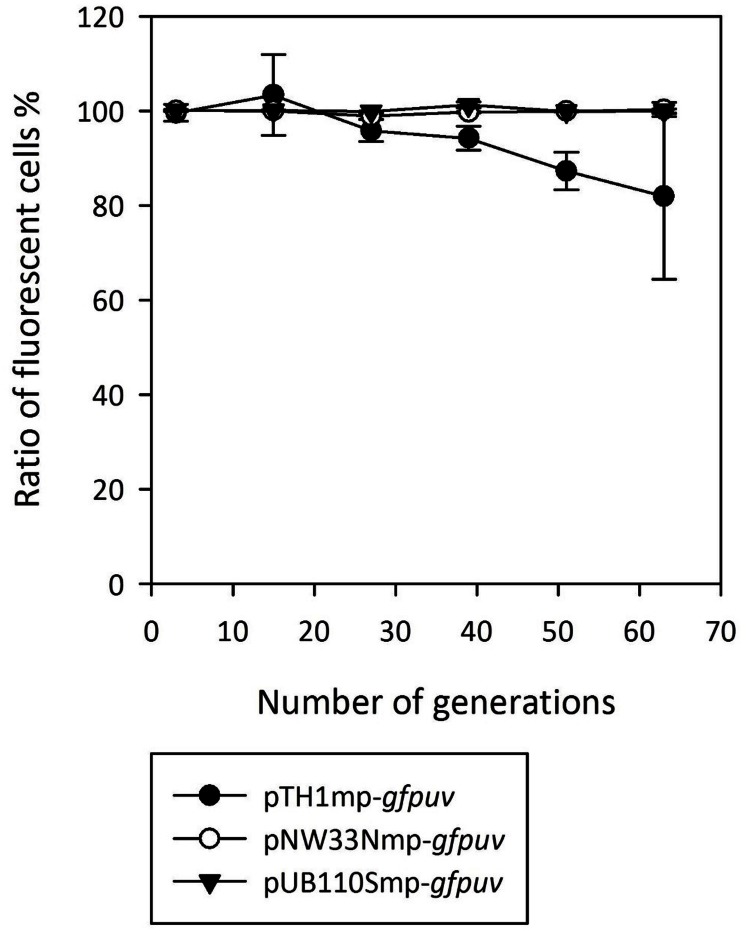
**Stability of *gfpuv* gene expression based on the RC plasmids used in the study**. The graph presents the ratio of the fluorescent cells to all the cells analyzed during growth in medium in presence and absence of antibiotics for 60 generations. Means of triplicates with standard deviation are shown.

Next, we compared the plasmid stability for *gfpuv*-expressing RC plasmids transferred to *B. methanolicus* MGA3. The strains were grown for 60 generations in media with and without antibiotic selection and plasmid-containing cells emitting a fluorescence signal were counted every 12 generations. As shown in **Figure 1** only the pTH1mp plasmid was lost at a significant level over the course of the experiment.

### Cadaverine Production from Methanol by Expression of a Heterologous Lysine Decarboxylase Gene from a Theta-Replicating Plasmid

The plasmids pTH1mp and pBV2mp, containing the *mdh* promoter were used to study cadaverine production in *B. methanolicus* during fed-batch methanol fermentation. We have previously reported a methanol-based cadaverine production titer of 6.5 g/L by *B. methanolicus* MGA3 (pTH1mp-*cadA*), a strain overexpressing the lysine decarboxylase *cadA* gene from *E. coli* (corrigendum to Nærdal et al., 2015). We compared cadaverine production in the strain overexpressing *cadA* from a theta-replicating plasmid during high cell density fed-batch fermentation. The *B. methanolicus* strain MGA3 (pBV2mp-*cadA*) was tested in duplicates under comparable fermentation conditions. Samples for cadaverine and amino acid analysis, cell dry weight and OD_600_ were taken throughout the cultivation. As presented in **Table 2**, we obtained a cadaverine production titer of 10.2 g/L based on the alternative theta-replicating pBV2mp plasmid. A substantial 55% production increase compared to the previously reported (pTH1mp-*cadA*)-based strain was observed. While biomass and by-product levels were similar between the two strains, the specific growth rate of MGA3 (pBV2mp-*cadA*) was lower than that of MGA3 (pTH1mp-*cadA*) (**Table 2**).

**Table 2:** Fed-batch methanol fermentation production data of strains MGA3 (pBV2mp-*cadA*) and MGA3 (pTH1mp-*cadA*).

Strain	CDW^a^	μ^b^	Asp^c^	Glu^c^	Ala^c^	Lys^c^	Cad^c^
	
	g/L	h^-1^	g/L	g/L	g/L	g/L	g/L
MGA3 (pBV2mp-*cadA*)	60.9	0.38	1.6	72.2	9.2	0.5	10.2
MGA3 (pTH1mp-*cadA*)	65.5	0.45	1.5	71.8	10.2	0.0	6.5

*Mean values of duplicate cultures for *B. methanolicus* MGA3 (pBV2mp-*cadA*) are shown. Deviation did not exceed 10%. The MGA3 (pTH1mp-*cadA*) data was imported from Nærdal et al. (2015). CDW, cell dry weight; μ, specific growth rate; Asp, L-aspartate; Glu, L-glutamate; Ala, L-alanine; Lys, L-lysine; Cad, cadaverine. ^a^Biomass concentrations are maximum values from the stationary growth phase. ^b^Specific growth rates are maximum values calculated from the exponential growth period. ^c^Cadaverine and amino acid concentrations are maximum values and volume corrected*.

### Plasmid Compatibility

**FIGURE 2:**
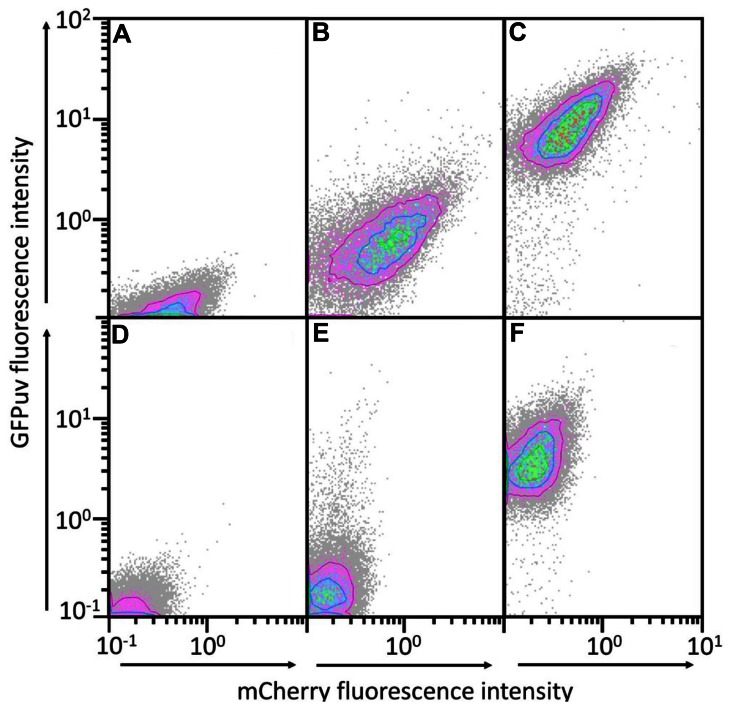
**GFPuv and mCherry fluorescence contour plots of populations of *B. methanolicus* MGA3 transformed with two gene expression plasmids**. mCherry and GFPuv fluorescence of *B. methanolicus* MGA3 (pTH1mp-*mcherry*) **(A)**, MGA3 (pTH1mp-*mCherry*)(pBV2mp-*gfpuv*) **(B)**, MGA3 (pTH1mp-*mCherry*)(pUB110Smp-*gfpuv*) **(C)**, MGA3 wild type **(D)**, MGA3 (pBV2mp-*gfpuv*) **(E)**, and MGA3 (pUB110Smp-*gfpuv*) **(F)**. mCherry fluorescence intensities are depicted on the x axis and GFPuv fluorescence intensities on the y axis. Contour plots of populations of 20000 cells are depicted.

In order to establish a two plasmid-based gene expression system, we analyzed the compatibility of the chosen RC (pTH1mp and pUB110Smp)- and theta (pBV2mp)-replicating plasmids in *B. methanolicus*. pTH1mp and pUB110Smp share high identity (42%) of their replication protein Rep and of the origin of replication sequence (95%) and for this reason it was not clear whether they can coexist in the same cell. Similarly, we did not analyze the pUB110mp/pNW33N plasmid pair which display 100% identity of Rep protein sequences. Plasmids for expression of either *gfpuv* or *mcherry* (Shaner et al., 2004) were constructed to simultaneously analyze gene expression from two vectors. The following plasmid combinations were applied: pTH1mp-*mcherry* with pUB110Smp-*gfpuv* or pTH1mp-*mcherry* with pBV2mp-*gfpuv*. Overexpression of *mcherry* from pTH1mp led to red fluorescence (depicted on the y-axis in **Figure 2**). Similarly, overexpression of *gfpuv* from pUB110Smp or from pBV2mp yielded green-fluorescent cells (x-axis of **Figure 2**). Cells transformed with pTH1mp-*mcherry* and pUB110Smp-*gfpuv* or with pTH1mp-*mcherry* and pBV2mp-*gfpuv* showed simultaneous red and green fluorescence (**Figure 2**) providing evidence for two plasmid-based gene expression in *B. methanolicus*.

### Construction of Mannitol Inducible Gene Expression System

**FIGURE 3:**
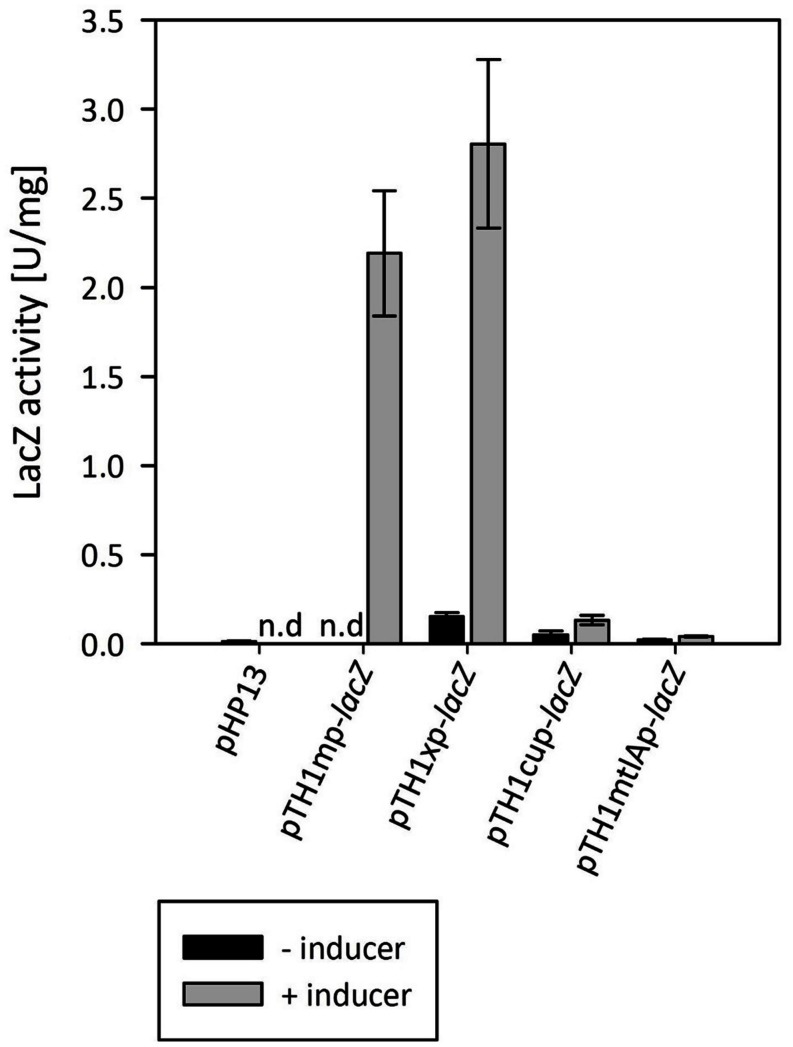
**Screening of various inducible gene expression systems with *lacZ* used as reporter gene**. The methanol, xylose, mannitol and copper inducible systems were compared. Means of triplicates with standard deviation are shown, n.d – no data.

In order to choose a suitable system for inducible gene expression we screened several inducible promoter systems using the thermostable LacZ from *B. coagulans* as a reporter (Kovács et al., 2010). We have tested the *B. megaterium* xylose inducible system from plasmid pHCMC04 (Nguyen et al., 2005), a native mannitol inducible promoter from MGA3, and a copper inducible promoter from *Lactobacillus sakei* (Crutz-Le Coq and Zagorec, 2008). As shown in **Figure 3**, the xylose inducible promoter system was functional in *B. methanolicus* MGA3 and, when fully induced, yielded higher expression levels than the hitherto used *mdh* promoter. Very low expression was observed from both the mannitol-inducible promoter present in the upstream region of the *mtlA* gene of *B. methanolicus* MGA3, and the copper inducible promoter. The copper-inducible promoter showed a dose-response where the activity in cultures induced by 100 μM CuSO_4_ was approximately threefold higher than in cultures induced by 50 μM CuSO_4_ (data not shown). The inducer, however, had a toxic effect on the cells, reducing the growth rate considerably at concentrations above 50 μM CuSO_4_ (data not shown), making it not suitable for industrial applications.

**FIGURE 4:**
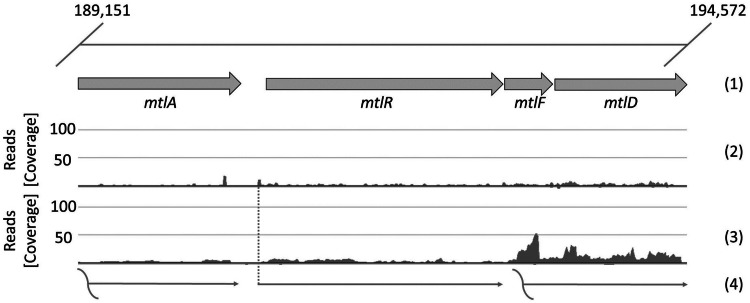
**Transcriptional organization of mannitol utilization pathway genes of *B. methanolicus* MGA3**. The first line (1) represents the genomic organization of the genes in the cluster, the next one (2) the mapped reads of 5′-ends of primary transcripts, the third (3) depicts the mapped reads of whole primary transcripts and the last (4): the putative corresponding transcripts. The level of transcription is visualized with ReadXplorer (Hilker et al., 2014) and given as absolute reads (coverage) at the corresponding genomic positions. Data are based on the RNA-seq analysis of Irla et al. (2015).

Since only very low expression was observed from the mannitol-inducible *mtlA* promoter, we used DNA microarray data (Heggeset et al., 2012) and RNA-seq data (Irla et al., 2015) to identify other mannitol-inducible genes. **Figure 4** presents the genomic and transcriptomic organization of four genes which belong to the mannitol utilization pathway: *mtlA* coding for PTS system mannitol-specific EIICB component, *mtlR* encoding a transcriptional regulator, *mtlF* coding for mannitol-specific phosphotransferase enzyme IIA and *mtlD* encoding mannitol-1-phosphate 5-dehydrogenase. Genes *mtlF* and *mltD* are co-expressed as an operon. Transcription start sites (TSSs) were not detected either for *mtlA* or for *mtlF*-*mtlD*; however, a TSS was found for *mtlR* (Irla et al., 2015). This 5′ untranslated region (5′ UTR) of *mtlR* is 80 nt in length and its upstream sequence contains conserved -10 and -35 regions (bold): 5′-**TTGTAT**TAAGGGATATAAACGTTT**TATGAT**AAATATG-3′, furthermore the putative ribosome binding site (RBS) sequence is AGTGGAG, which differs in two positions from the *B. methanolicus* consensus RBS motif AGGAGG (Irla et al., 2015). We cloned the upstream sequence of the *mtlR* gene into the plasmid pTH1 containing the *gfpuv* gene and exchanged the RBS sequence to the consensus motif, which resulted in plasmid pTH1m2p-*gfpuv*.

**FIGURE 5:**
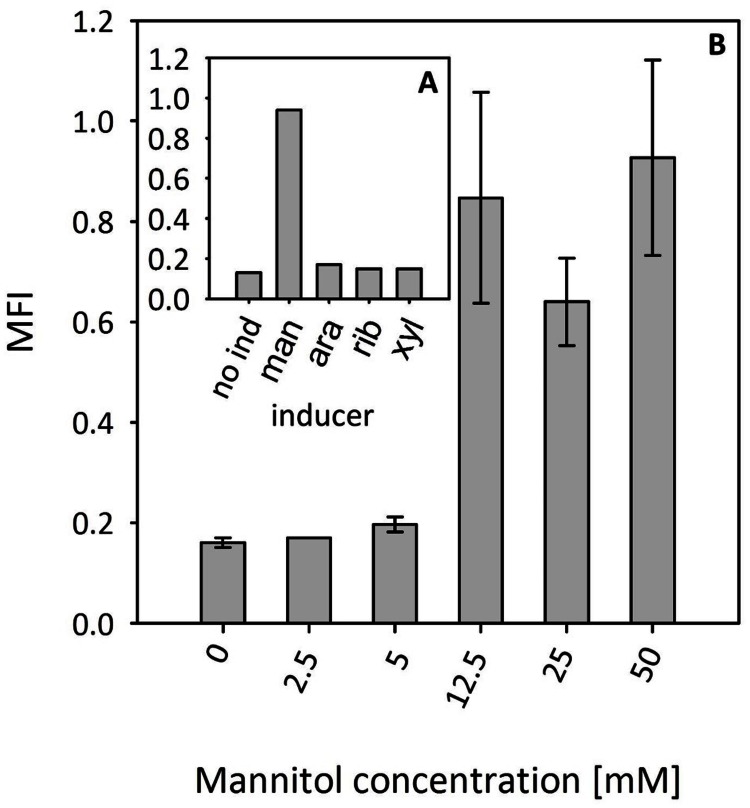
**Specificity and dynamic range of induction of the *B. methanolicus mtlR* promoter**. **(A)** Screening of sugar alcohols as potential inducers. Single replicates of shake flask cultures are presented. **(B)** Expression titration with different mannitol concentrations. Abbreviations: no ind, no inducers; man, mannitol; ara, arabitol; rib, ribitol; xyl, xylitol; MFI, median fluorescence intensity of GFPuv given in a.u. GFPuv fluorescence of exponentially growing cells was measured after 2 h incubation at 37°C and 200 rpm Mean values and standard deviations of triplicate shake flask cultures are presented.

At first, several sugar alcohols were tested as potential inducers (**Figure 5A**). Supplementation with 50 mM mannitol induced *gfpuv* expression; however, neither arabitol, ribitol, nor xylitol induced reporter gene expression (**Figure 5A**). Subsequently, a titration experiment with different concentrations of mannitol was performed. While the addition of 5 mM mannitol did not increase reporter gene expression, high GFPuv fluorescence intensities were observed upon addition of 12.5, 25, and 50 mM mannitol (**Figure 5B**). It has to be noted that GFPuv fluorescence intensities in the presence of 12.5, 25, and 50 mM mannitol were comparable suggesting that full induction has been achieved (**Figure 5B**). The expression level of the mannitol-inducible promoter in the presence of 50 mM mannitol was similar to that obtained with the conventionally used *mdh* promoter. To test whether higher gene expression is possible in the mannitol inducible system, we decided to exchange the sequences of the -10 and/or the -35 region for the previously described consensus sequences (Irla et al., 2015). As shown in **Table 3**, the exchange of the -35 region or the -35 region together with the -10 region led to higher fluorescence levels in comparison to the native promoter. However, the double exchange caused a 3.5-folds increased background expression.

**Table 3:** Reporter gene expression from the *Bacillus methanolicus* mannitol inducible *mtlR* promoter with changed -35 and -10 region sequences.

Promoter name	-35 region	-10 region	Median fluorescence intensity of GFPuv [a.u.]	
			0 mM mannitol	50 mM mannitol
m2p	TTGTAT	TATGAT	0.2 ± 0.0	1.1 ± 0.2
m21p	---A-A	------	0.2 ± 0.0	2.9 ± 0.1
m22p	---A-A	---A--	0.6 ± 0.1	3.8 ± 0.3
m23p	------	---A--	0.2 ± 0.0	0.9 ± 0.4

*GFPuv fluorescence of exponentially growing cells was measured after 2 h incubation at 37°C and 200 rpm. Mean values and standard deviations of triplicate shake flask cultures are given*.

**FIGURE 6:**
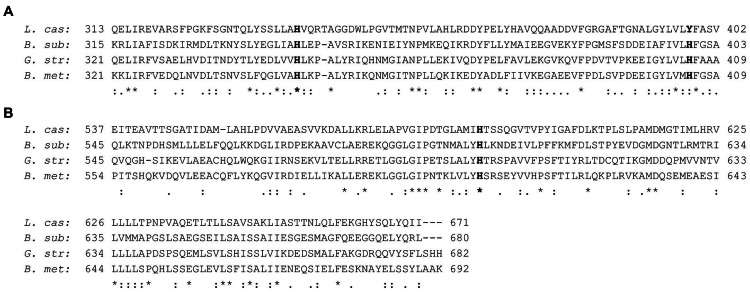
**Amino acid sequence alignment of the PRD2 **(A)** and EII^Mtl^**(B)** domains of various MtlR proteins**. The known regulatory sites are in boldface, conserved sequence (^∗^), conservative mutations (:), semi-conservative mutations (.), and non-conservative mutation ( ). The alignment was performed with T-Coffee (Notredame et al., 2000). The GenBank accession numbers of the sequences are as follows: *L. cas*: *Lactobacillus casei* BL23, FM177140.1; *B. sub*: *B. subtilis* ssp. *subtilis* str. 168, CP010052.1; *G. str: Geobacillus stearothermophilus* ATCC 7954, U18943.1; *B. met*: *B. methanolicus* MGA3, CP007739.1.

**FIGURE 7:**
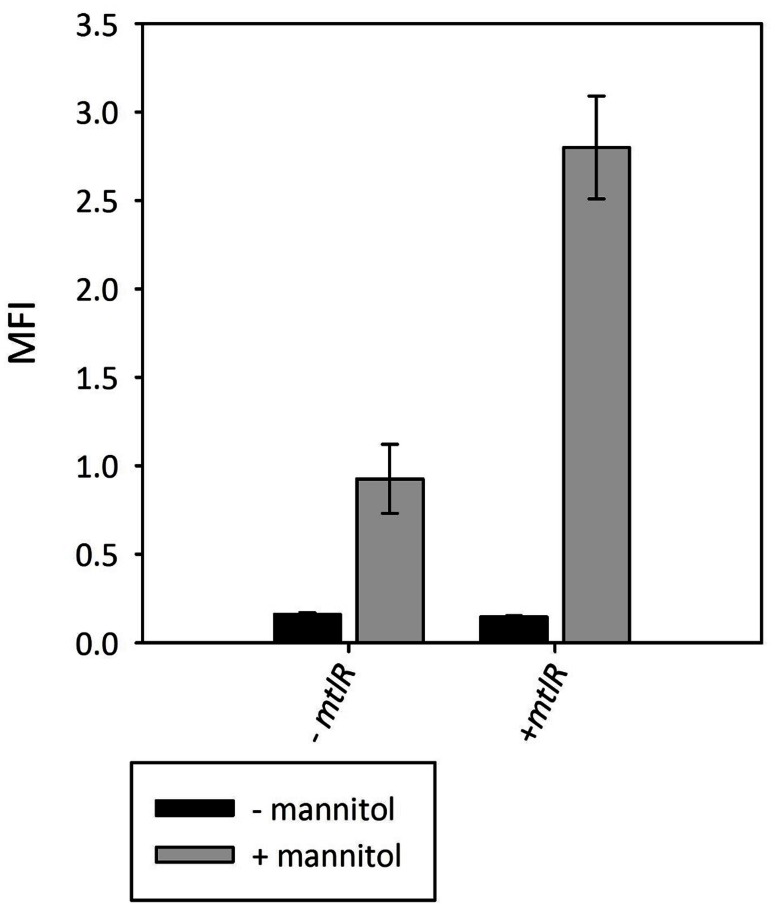
**Effect of plasmid-borne overexpression of *mtlR* on mannitol-inducible *gfpuv* reporter gene expression from *mtlR* promoter variant m2p**. MFI - median fluorescence intensity of GFPuv given in a.u. GFPuv fluorescence of exponentially growing cells was measured after 2 h incubation at 37°C and 200 rpm. Mean values and standard deviations of triplicate shake flask cultures are given.

MtlR has been characterized as a mannitol-dependent transcriptional activator in several species (Joyet et al., 2015). The alignment of the *B. methanolicus* MtlR protein sequence with the sequences of characterized regulators from *L. casei* BL23, *B. subtilis* ssp. *subtilis* str. 168 and *Geobacillus stearothermophilus* ATCC 7954 (**Figure 6**) revealed the conserved residues important for the regulatory activity of MtlR. The high similarity to the characterized proteins suggested that MtlR of *B. methanolicus* most probably serves as a transcriptional activator. For this reason, we decided to test whether the plasmid-borne overexpression of *mtlR* increased *mtlR* promoter activity. As shown in **Figure 7**, the overexpression of this gene increased the reporter gene expression from the mannitol inducible promoter m2p by more than 2.5-fold while maintaining a low level of background expression in the absence of mannitol. Taken together, evidence is provided for a versatile mannitol inducible system for the thermophilic *B. methanolicus* on the basis on the previously obtained RNA-seq data.

### Xylose Inducible Gene Expression in *B. methanolicus*

**FIGURE 8:**
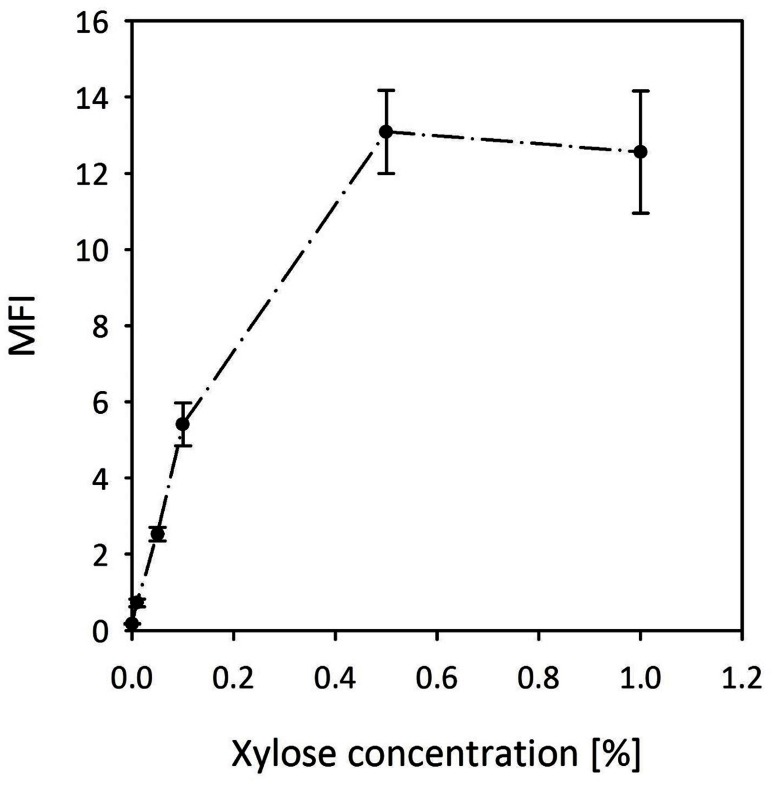
**Induction of reporter gene expression from the xylose inducible promoter of pTH1xpx-*gfpuv***. MFI - median fluorescence intensity of GFPuv given in a.u. GFPuv fluorescence of exponentially growing cells was measured after 2 h incubation at 37°C and 200 rpm. Mean values and standard deviations of triplicate shake flask cultures are presented.

Based on our screening experiments (**Figure 3**), we decided to further develop the xylose inducible system for gene expression in *B. methanolicus*. We have subcloned the *xylR* regulator gene together with the promoter and the RBS sequence of the *B. megaterium xylA* gene into pTH1 to drive expression of *gfpuv*. The resulting plasmid was named pTH1xpx-*gfpuv*. **Figure 8** shows the expression levels of *gfpuv* transcribed from the xylose inducible promoter in media with different xylose concentrations. Reporter gene expression increased linearly in the concentration range between 0.01% (w/v) and 0.1% (w/v) and reached a plateau at 0.5% (w/v). The fluorescence from fully induced xpx promoter is around 15-fold higher in comparison to the conventionally used *mdh* promoter (**Table 1**). Furthermore, the background gene expression with uninduced MGA3 (pTH1xpx-*gfpuv*) was very low (0.17 ± 0.01 a.u.) as compared to the background fluorescence (0.12 ± 0.00 a.u.) obtained for wild type *B. methanolicus* MGA3. Notably, mannitol did not induce expression of *gfpuv* from the xylose inducible promoter (data not shown).

### Introduction of Heterologous Starch Degradation Pathway in *B. methanolicus* MGA3 by Heterologous Overexpression of α-Amylase Gene from *Streptomyces griseus* IMRU3570

**FIGURE 9:**
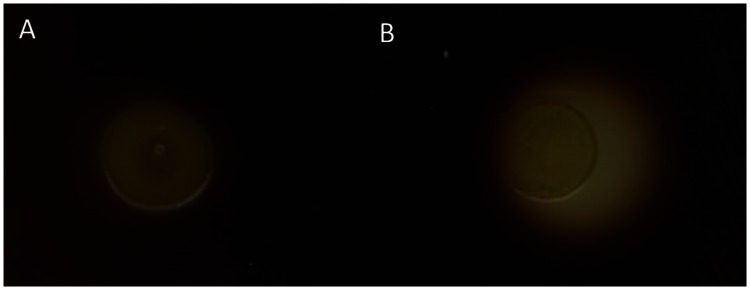
**Starch degradation by *B. methanolicus* strains**. The strains were grown on LB plates supplemented with 0.5% starch and 0.05% xylose and iodine added to detect starch degradation. The dark area in the plate indicates presence of starch and the colorless halo around cells indicates starch degradation. **(A)** *B. methanolicus* MGA3 (pTH1mp) and **(B)** *B. methanolicus* MGA3 (pTH1xpx-*amy*).

α-Amylases degrade starch to glucose and expression of heterologous α-amylase genes in glucose-positive, but starch-negative species enabled starch utilization as for example shown for *C. glutamicum* expressing α-amylase gene (*amy*) from *Streptomyces griseus* IMRU3570 (Seibold et al., 2006). A BLAST search of the *Bacillus methanolicus* genome revealed two genes putatively encoding α-amylases (BMMGA3_04340, BMMGA3_04345) and one coding for an α-glucosidase (Heggeset et al., 2012; Irla et al., 2014). For heterologous expression of *amy* from *S. griseus* plasmid pTH1xpx was used for xylose inducible expression in *B. methanolicus*. Starch degradation by the control strain *B. methanolicus* MGA3 (pTH1mp) on LB agar plates supplemented with 0.5% soluble starch and 0.05% xylose at 37°C was not observed (**Figure 9A**). By contrast *B. methanolicus* MGA3 (pTH1xpx-*amy*) showed a halo on starch LB plates containing xylose as an inducer and incubated at 37°C (**Figure 9B**) indicating that expression of *amy* from *S. griseus* plasmid allowed for starch degradation by recombinant *B. methanolicus*.

## Discussion

In this study we have developed a versatile toolbox for inducible gene expression in *B. methanolicus* from RC- and theta-replicating plasmids. As a test case, we have applied a theta-replicating plasmid for heterologous expression of the lysine decarboxylase gene from *E. coli* and have shown improved cadaverine (1,5-diaminopentane) production in methanol-controlled fed-batch fermentations.

Strain development for *B. methanolicus* until recently relied on (over-)expression of genes or operons from a single plasmid despite the need for gene co-expression from two different plasmids and for inducible gene expression (Brautaset et al., 2010; Nærdal et al., 2015). To that end, we have extended the existing portfolio of available expression vectors (based on pHP13 and pNW33N) with the two additional replicons pUB110 and pHCMC04 (Gryczan et al., 1978; Cue et al., 1997; Nguyen et al., 2005; Nilasari et al., 2012). Plasmids pUB110, pHP13 and pNW33N replicate via a RC mechanism (Khan, 1997) and belong to the same plasmid family. This family is named pC194/pUB110 and is characterized by a similar *ori* sequence CTT(G)TTCTTTCTTATCTTGATA. However, they are known to have different copy numbers in *B. subtilis*. Typically, RC plasmids are known to replicate in thermophilic bacteria (Soutsehek-Bauer et al., 1987; Cue et al., 1997; Rhee et al., 2007). This, to the best of our knowledge, was not known for theta-replicating plasmids and we show here for the first time that the theta-replicating plasmid pHCMC04 replicates stably in the thermophilic *B. methanolicus.*

Cadaverine production by recombinant *B. methanolicus* expressing the *E. coli* lysine decarboxylase gene *cadA* was superior when using the theta-replicating plasmid pBV2mp-*cadA* (pHCM04 replicon) as compared to the rolling circle-replicating plasmid pTH1mp-*cadA* (pHP13 replicon) (**Table 2**). Despite the low copy number of the pHCMC04 replicon (approximately half of that for replicon pHP13) confirmed both by ddPCR and GFPuv fluorescence measurement (**Table 1**), cadaverine production by *B. methanolicus* MGA3 (pBV2mp-*cadA*) was about 55% higher (**Table 2**) than by MGA3 (pTH1mp-*cadA*). This observation results most probably from two factors: loss of the pHP13 replicon over cultivation time and high stability of the pHCMC04 replicon. The loss of the pHP13 replicon was somewhat surprising as it was reported to be stable in *B. methanolicus* (Cue et al., 1997). Plasmid pHP13 contains the *ori* sequence from parental plasmid pTA1060 (Haima et al., 1987), but is however lacking a 167-bp fragment outside of the *ori* sequence from pTA1060, which has been shown to improve stable plasmid segregation (Bron et al., 1987; Chang et al., 1987; Haima et al., 1987). By contrast, the theta-replicating plasmid pHCMC04 showed the expected high stability typically observed for this type of plasmids (Bruand et al., 1991; Titok et al., 2003; Nguyen et al., 2005). High plasmid stability is important in large-scale industrial processes requiring long seed trains or in fed-batch and continuous cultivations since subpopulations of cells which have lost plasmids due to low segregational stability usually lead to significant productivity losses (Friehs, 2004). With respect to methanol utilization in the fed-batch cultivations, it was observed that the overall carbon consumption (from methanol) of strains MGA3 (pTH1mp-*cadA*) and MGA3 (pBV2mp-*cadA*) differed by less than 5% (data not shown). Thus, the finding that cadaverine production increased by 6.2 g/L and biomass formation was reduced by 4.6 g/L (**Table 2**), suggested a reallocation of carbon source utilization from biomass to product formation.

Here we have characterized the plasmid pUB110 as a very feasible choice for gene expression in thermophilic *B. methanolicus* for two reasons: it showed the highest copy number among the tested replicons (**Table 1**) and showed high segregational stability (**Figure 1**). In *B. subtilis*, the pUB110 plasmid is known as a high copy number plasmid (Gryczan et al., 1978), whereas segregational stability seems to be a more complex issue. It was shown in several studies that the wild type plasmid is stable over multiple generations in different *Bacillus* spp. including *B. subtilis* (Polak and Novic, 1982; Alonso et al., 1987; Shoham and Demain, 1990), *B. thuringiensis* (Naglich and Andrews, 1988) and *B. sphaericus* (Seyler et al., 1991). Nonetheless, molecular modifications may lead to decreased stability of pUB110 for several different reasons. The segregational instability may be the function of the insert size (Bron et al., 1988; Zaghloul et al., 1994) or the high expression level of the cloned gene (Vehmaanperii and Korhola, 1986). Moreover, the lack of the so-called BA3 and BA4 regions has been described to destabilize the plasmid (Tanaka and Sueoka, 1983; Bron and Luxen, 1985; Shoham and Demain, 1990). Despite the fact that the pUB110-derived plasmid (pUB110Smp) used in this study did not contain BA3 and BA4 sequences and contained a 2.4 kbp insert, it was stable over 60 generations in *B. methanolicus* (**Figure 1**). Taken together, in thermophilic *B. methanolicus* pUB110Smp seems to be more feasible for molecular cloning than the hitherto used pTH1mp replicon.

Additionally, pUB110Smp as well as the theta-replicating pBV2mp were shown to be compatible with pTH1mp and could be used for independent expression of two genes in a two-plasmid approach.

*Bacillus methanolicus* not only grows with mannitol as the carbon source, but also shows mannitol dependent induction of at least two promoters P*_mtlA_* and P*_mtlR_* as revealed by transcriptome and proteome analyses (Heggeset et al., 2012; Müller et al., 2014; Irla et al., 2015). As compared to growth on methanol, mannitol-grown cells showed about 20-fold higher abundances of the proteins involved in mannitol utilization, i.e., EIIA and EIIBC components of the mannitol-specific PTS and mannitol-1-phosphate 5-dehydrogenase (Müller et al., 2014). The genomic organization suggests monocistronic transcription of *mtlA* and co-transcription of *mtlRFD* although RNA-seq data also suggest co-transcription of *mtlFD* without *mtlR* (see **Figure 2**). Reporter gene expression from P*_mtlR_* was higher than from P*_mtlA_*. Background expression from P*_mtlR_* was low and the induction when grown in the presence of mannitol was 6.5-fold for the native promoter and 13-fold for the improved version. These values are lower compared to mannitol inducible promoters in *B. subtilis* and *Pseudomonas putida* which show induction rates of about 20 for the native promoters and up to 176 for modified versions (Heravi et al., 2011; Hoffmann and Altenbuchner, 2015). The threshold concentration of mannitol required for induction of the promoter *mtlR* in the vector pTH1m2p was about 12.5 mM which is higher than the Monod constant, i.e., the concentration supporting growth with mannitol with a half-maximal growth rate (about 0.5 mM). This different threshold may reflect basal expression of mannitol utilization genes (e.g., *mtlA, mtlFD* operon). Moreover, we used the promoter of the regulatory gene *mtlR* rather than a promoter of a structural gene (e.g., *mtlA*, *mtlFD* operon) and dose dependency of induction of *mtlA* or the *mtlFD* operon might differ from dose dependency of induction of *mtlR*. Thus, it is conceivable that when mannitol is present in limited concentrations in the environment the background expression of the mannitol utilization genes is sufficient for initial mannitol utilization, whereas only higher mannitol concentrations lead to autoinduction of *mtlR* expression.

Induction of P*_mtlR_* was specific to mannitol, while similar sugar alcohols did not affect transcription. The fact that mannitol is one of the few carbon sources of *B. methanolicus* precludes its use as gratuitous inducers similar to IPTG in the *E. coli lac* system or the xylose system applied to *B. methanolicus* (see below).

The xylose inducible system originating from *B. megaterium* was previously successfully used in several bacterial species, including *B. megaterium* (Rygus and Hillen, 1991), *B. subtilis* (Kim et al., 1996), *Staphylococcus aureus* (Zhang et al., 2000), and *Brevibacillus choshinensis* (D’Urzo et al., 2013). Here we show that this system also works in *B. methanolicus*. Since xylose is not metabolized by *B. methanolicus* it serves as a gratuitous inducer in this bacterium. In fact, the xylose inducible system turned out to have multiple advantages, including very low background expression in the uninduced state, titratable induction, and a 75-fold induction window between the uninduced and the fully induced state. Similar high dynamic ranges of xylose induction have been reported for other *Bacillus* ssp. (Kim et al., 1996; Zhang et al., 2000; Bhavsar et al., 2001). However, catabolite repression of the xylose inducible promoter in multiple *Bacillus* ssp. is disadvantageous for biotechnological applications. Catabolite repression is due to the *cis*-acting catabolite responsive element (*cre*), which is a binding site of the catabolite repressor protein CcpA (Jacob et al., 1991; Lokman et al., 1994; Kim et al., 1996; Schmiedel and Hillen, 1996; Chaillou et al., 1998; Bhavsar et al., 2001; Miyoshi et al., 2004). To avoid this phenomenon, the *cre* sequence TGAAAGCGCAAACA of the *xyl* operon in *B. megaterium*, which is located within the of *xylA* gene (Schmiedel and Hillen, 1996), is not present in the plasmids used here. The absence of the *cre* sequence from the plasmids may be relevant since the genome of *B. methanolicus* encodes a homolog of CcpA (BMMGA3_13325). A BLAST search did not indicate that the *cre* sequence TGAAAGCGCAAACA is present upstream of the genes for carbon source utilization (only methanol, glucose, or mannitol are known carbon sources) of *B. methanolicus*. A *cre* sequence may be present upstream of the putative glucosamine-6-phosphate synthetase encoding gene *glmS* (BMMGA3_01020). In *B. subtilis, glmS* mRNA acts as a metabolite-responsive ribozyme (Winkler et al., 2004) and glucose-repressive *glmS* transcription is at least partially under CcpA-independent control (Yoshida et al., 2001). However, the regulatory mechanism of the homolog of CcpA (BMMGA3_13325) of *B. methanolicus* and its target genes need still to be defined. Plasmid pTH1xpx for xylose inducible gene expression was applied for heterologous expression of *Streptomyces griseus*-derived α-amylase gene in *B. methanolicus.* As shown before for mesophilic *Corynebacterium glutamicum* (Seibold et al., 2006), heterologous expression of the α-amylase gene from *S. griseus* supported starch degradation by recombinant *B. methanolicus* assayed at 37°C. While α-amylase from *S. griseus* was an obvious choice it has its limitations in thermophiles since the enzyme is known to exhibit maximal activity at 30°C with 92% of the remaining activity at 40°C, but only trace activity being observable at 50°C (Simpson and McCoy, 1953). Nonetheless, heterologous expression of *amy* from *S. griseus* serves as an example that the gene expression tools described here are suitable for pathway engineering of *B. methanolicus.*

Taken together, a series of plasmids for stable replication in the thermophilic *B. methanolicus* was developed for xylose as well as mannitol inducible gene expression. Thus, an important step for further advancing this thermophilic bacterium as a very promising candidate for industrial production of amino acids and their derivatives has been reached. Improved production of cadaverine using a theta-replicating plasmid for heterologous expression of the lysine decarboxylase gene from *E. coli* in methanol-controlled fed-batch fermentations was demonstrated as a first application example, starch degradation by recombinant *B. methanolicus* carrying xylose inducible expression plasmid pTH1xpx with the gene for α-amylase from *S. griseus* as a second example.

## Author Contributions

MI, TH, IN, LP, TH, SL carried out the experimental procedure and the data analysis of the present study. MI prepared a draft of the manuscript. MI, TH, IN, SL, TB, and VW finalized the manuscript. TB and VW coordinated the study. All authors read and approved the manuscript.

## Conflict of Interest Statement

The authors declare that the research was conducted in the absence of any commercial or financial relationships that could be construed as a potential conflict of interest.
