# Corrigendum: Genome-Based Genetic Tool Development for *Bacillus methanolicus*: Theta- and Rolling Circle-Replicating Plasmids for Inducible Gene Expression and Application to Methanol-Based Cadaverine Production

**DOI:** 10.3389/fmicb.2019.00425

**Published:** 2019-03-12

**Authors:** Marta Irla, Tonje M. B. Heggeset, Ingemar Nærdal, Lidia Paul, Tone Haugen, Simone B. Le, Trygve Brautaset, Volker F. Wendisch

**Affiliations:** ^1^Genetics of Prokaryotes, Faculty of Biology and CeBiTec, Bielefeld University, Bielefeld, Germany; ^2^SINTEF Materials and Chemistry, Department of Biotechnology and Nanomedicine, Trondheim, Norway; ^3^Department of Biotechnology, Norwegian University of Science and Technology, Trondheim, Norway

**Keywords:** *Bacillus methanolicus*, thermophile, methylotroph, genetic tool box, theta-replicating plasmids, gene expression

In the original article, there was a mistake in Table 2 as published. The cadaverine concentrations reported were miscalculated and overestimated by 41.6%, because the HPLC standard solution consisted of “cadaverine dihydrochloride (175.1 g/mol)” and not “cadaverine (102.18 g/mol).” The corrected Table 2 appears below.

**Table 2:** Fed-batch methanol fermentation production data of strains MGA3(pBV2mp-*cadA*) and MGA3 (pTH1mp-*cadA*).

**Strain**	**CDW^a^**	**μ^b^**	**Asp^c^**	**Glu^c^**	**Ala^c^**	**Lys^c^**	**Cad^c^**
	**g/L**	**h^−1^**	**g/L**	**g/L**	**g/L**	**g/L**	**g/L**
MGA3(pBV2mp-*cadA*)	60.9	0.38	1.6	72.2	9.2	0.5	10.2
MGA3(pTH1mp-*cadA*)	65.5	0.45	1.5	71.8	10.2	0.0	6.5

*Mean values of duplicate cultures for B. methanolicus MGA3(pBV2mp-cadA) are shown. Deviation did not exceed 10%.The MGA3(pTH1mp-cadA) data was imported from Nærdal et al. (2015). CDW, cell dry weight; μ, specific growth rate; Asp, l-aspartate; Glu, l-glutamate; Ala, l-alanine; Lys, l-lysine; Cad, cadaverine*.

a *Biomass concentrations are maximum values from the stationary growth phase*.

b *Specific growth rates are maximum values calculated from the exponential growth period*.

c *Cadaverine and amino acid concentrations are maximum values and volume corrected*.

Because of the error reported above, a correction has been made to the **Abstract**:

“*Bacillus methanolicus* is a thermophilic methylotroph able to overproduce amino acids from methanol, a substrate not used for human or animal nutrition. Based on our previous RNA-seq analysis a mannitol inducible promoter and a putative mannitol activator gene *mtlR* were identified. The mannitol inducible promoter was applied for controlled gene expression using fluorescent reporter proteins and a flow cytometry analysis, and improved by changing the −35 promoter region and by co-expression of the *mtlR* regulator gene. For independent complementary gene expression control, the heterologous xylose-inducible system from *B. megaterium* was employed and a two-plasmid gene expression system was developed. Four different replicons for expression vectors were compared with respect to their copy number and stability. As an application example, methanol-based production of cadaverine was shown to be improved from 6.5 to 10.2 g/L when a heterologous lysine decarboxylase gene *cadA* was expressed from a theta-replicating rather than a rolling-circle replicating vector. The current work on inducible promoter systems and compatible theta- or rolling circle-replicating vectors is an important extension of the poorly developed *B. methanolicus* genetic toolbox, valuable for genetic engineering and further exploration of this bacterium.”

Additionally, a correction has been made to the **Results, Cadaverine Production From Methanol by Expression of a Heterologous Lysine Decarboxylase Gene From a Theta-Replicating Plasmid:**

“The plasmids pTH1mp and pBV2mp, containing the *mdh* promoter were used to study cadaverine production in *B. methanolicus* during fed-batch methanol fermentation. We have previously reported a methanol-based cadaverine production titer of 6.5 g/L by *B. methanolicus* MGA3 (pTH1mp-*cadA*), a strain overexpressing the lysine decarboxylase *cadA* gene from *E. coli* (corrigendum to Nærdal et al., 2015). We compared cadaverine production in the strain overexpressing *cadA* from a theta-replicating plasmid during high cell density fed-batch fermentation. The *B. methanolicus* strain MGA3 (pBV2mp-*cadA*) was tested in duplicates under comparable fermentation conditions. Samples for cadaverine and amino acid analysis, cell dry weight and OD_600_ were taken throughout the cultivation. As presented in Table 2, we obtained a cadaverine production titer of 10.2 g/L based on the alternative theta-replicating pBV2mp plasmid. A substantial 55% production increase compared to the previously reported (pTH1mp-*cadA*)-based strain was observed. While biomass and by-product levels were similar between the two strains, the specific growth rate of MGA3 (pBV2mp-*cadA*) was lower than that of MGA3 (pTH1mp-*cadA*) (Table 2).”

Lastly, in the original article, the reference for “(Nærdal et al., 2015)” was incorrectly written as “Nærdal, I., Pfeifenschneider, J., Brautaset, T., and Wendisch, V. F. (2015). Methanol-based cadaverine production by genetically engineered *Bacillus methanolicus* strains. *Microb. Biotechnol*. 8, 342–350. doi: 10.1111/1751-7915.12257”.

It should be “Nærdal, I., Pfeifenschneider, J., Brautaset, T., and Wendisch, V. F. (2015). Methanol-based cadaverine production by genetically engineered *Bacillus methanolicus* strains. *Microb. Biotechnol*. 8, 342–350. doi: 10.1111/1751-7915.12257. *Microb. Biotechnol*. 2019, 12, 182-183”.

The authors apologize for these errors and state that this does not change the scientific conclusions of the article in any way. The original article has been updated.
