# Supplementary material for: Genome-Based Genetic Tool Development for Bacillus methanolicus: Theta- and Rolling Circle-Replicating Plasmids for Inducible Gene Expression and Application to Methanol-Based Cadaverine Production
Source: Front Microbiol. 2016 Sep 22;7:1481. doi: 10.3389/fmicb.2016.01481 (PMC5031790; doi:10.3389/fmicb.2016.01481)
Supplement: Supplementary file 1 [file Data_Sheet_1.PDF]

Supplementary material to

Genome-Based Genetic Tool Development for *Bacillus methanolicus*: Theta- and Rolling Circle-Replicating Plasmids for Inducible Gene Expression and Application to Methanol-Based Cadaverine Production

Marta Irla[1], Tonje M. B. Heggeset[2], Ingemar Nærdal[2], Lidia Paul[1], Tone Haugen[2], Simone B. Le[2], Trygve Brautaset[2,3], Volker F. Wendisch[1]*

[1]Genetics of Prokaryotes, Faculty of Biology & CeBiTec, Bielefeld University, Bielefeld, Germany
[2]SINTEF Materials and Chemistry, Department of Biotechnology and Nanomedicine, Trondheim, Norway
[3]NTNU, Norwegian University of Science and Technology, Department of Biotechnology, Trondheim, Norway

Supplementary tables

Table S1. Bacterial strains used in this study.

Plasmid	Relevant characteristics	Reference
Escherichia coli		
DH5α	General cloning host, F-*thi*-1 *endA*1 *hsdR*17(r-,m-) *supE*44 _*lacU*169 (_80*lacZ*_M15) *recA*1 *gyrA*96 *relA*1	Stratagene
MG1655	Wild type strain	ATCC 47076
Bacillus methanolicus		
MGA3	Wild type strain	ATCC 53907

Table S2. Plasmids used and constructed in this study.

Plasmid	Relevant characteristics	Reference
pHP13	Cm^R and Em^R; *E. coli*/*Bacillus* spp. shuttle vector	(Haima et al., 1987)
pTH1mp-*lysC*	Cm^R; derivative of pHP13 for expression of *lysC* under control of the *mdh* promoter (*mp*)	(Brautaset et al., 2010)
pUB110	Km^R; *Bacillus* spp. expression vector	(Gryczan et al., 1978)
pNW33N	Cm^R; *E. coli*/*Bacillus* spp. shuttle vector	(Rhee et al., 2007)
pHCM04	Cm^R and Ap^R; *E. coli*/*Bacillus* spp. shuttle vector for xylose controlled gene expression	(Nguyen et al., 2005)
pNZlacZ	Cm^R; *E. coli/*Bacillus spp. shuttle vector encoding the thermostable *B. coagulans* DSM 1 *lacZ*	(Kovács et al., 2010)
pRV613	Em^R and Ap^R; *E. coli*/*Lactobacillus* shuttle vector encoding the cupper-inducible P_{atkY}::*atkY* promoter system	(Crutz-Le Coq and Zagorec, 2008)
pUCG18	Km^R; *E. coli/Geobacillus* spp. shuttle vector	(Taylor et al., 2008)
pTH1xp	Cm^R; *E. coli*/*Bacillus* spp. shuttle vector for xylose inducible gene expression	This study
pTH1mp	Cm^R; derivative of pTH1mp-*lysC* for gene expression under control of the *mdh* promoter,	This study
pNW33Nmp	Cm^R; pNW33N derivative for gene under control of the *mdh* promoter	This study
pNW33Nkan	Cm^R, Km^R; pNW33N derivative in which the *knt*-resistance gene was inserted	This study
pBV2mp	Km^R and Ap^R; derivative of pHCM04 with xylose inducible promoter replaced by *mdh* promoter and *cat* replaced by *knt*	This study
pUB110Smp	Km^R; pUB110 derivative, *E. coli*/*Bacillus* spp. shuttle vector for gene under control of the *mdh* promoter	This study
pEPR1	Km^R; vector for resting *In vivo* promoter activities in *Corynebacterium glutamicum*; here used as source of *gfpuv* gene	(Knoppova et al., 2007)
pZ8-1_rfp	Km^R; source of *mCherry*	(Cleto et al., 2016)
pTH1mp-*gfpuv*	Cm^R; pTH1mp derivative for *gfpuv* expression under control of the *mdh* promoter	This study
pBV2mp-*gfpuv*	Km^R and Ap^R; pBVmp derivative for *gfpuv* expression under control of the *mdh* promoter	This study
pUB110Smp-*gfpuv*	Km^R; pUB110Smp derivative for *gfpuv* expression under control of the *mdh* promoter	This study
pNW33Nmp-*gfpuv*	Cm^R; pNW33Nmp derivative for *gfpuv* expression under control of the *mdh* promoter	This study
pTH1mp-*mcherry*	Cm^R; pTH1mp derivative for *mcherry* expression under control of the *mdh* promoter	This study
pTH1m2p-*gfpuv*	Cm^R; pTH1mp-*gfpuv* derivative for *gfpuv* expression under control of the mannitol inducible *mtlR* promoter (P_{mtlR})	This study
pTH1m21p-*gfpuv*	Cm^R; pTH1m2p-*gfpuv* derivative for *gfpuv* expression under control	This study

	of a mutated mannitol inducible promoter	
pTH1m22p-*gfpuv*	Cm^R; pTH1m21p-*gfpuv* derivative for *gfpuv* expression under control of a mutated mannitol inducible promoter	This study
pTH1m23p-*gfpuv*	Cm^R; pTH1m2p-*gfpuv* derivative for *gfpuv* expression under control of a mutated mannitol inducible promoter	This study
pTH1Pm2p-*gfpuv*	Cm^R; pTH1m21p-*gfpuv* derivative for *gfpuv* expression under control of the mannitol inducible *mtlR* promoter and *mtlR* expression under control of the *mdh* promoter	This study
pTH1xpx-*gfpuv*	Cm^R; pTH1xp derivative for *gfpuv* expression under control of the xylose inducible promoter	This study
pTH1mp-*lacZ*	Cm^R; pTH1mp-*lysC* derivative for *lacZ* expression under control of the *mdh* promoter	This study
pTH1mcs-*lacZ*	Cm^R; pTH1mp-*lacZ* derivative encoding a promoter-less *lacZ*	This study
pHCMC04-*lacZ*	Cm^R; pHCMC04 derivative for *lacZ* expression under control of the xylose inducible promoter	This study
pTH1xp-*lacZ*	Cm^R; pTH1mcs-*lacZ* derivative for *lacZ* expression under control of the xylose inducible promoter	This study
pTH1cup-*lacZ*	Cm^R; pTH1mcs-*lacZ* derivative for *lacZ* expression under control of the cupper inducible promoter P_{atkY}::*atkY*	This study
pTH1mtlAp-*lacZ*	Cm^R; pTH1mcs-*lacZ* derivative for *lacZ* expression under control of the mannitol inducible *mtlA* promoter (P_{mtlA})	This study
pTH1mp-*cadA*	Cm^R; pTH1mp derivative for *cadA* expression under control of the *mdh* promoter	(Nærdal et al., 2015)
pBV2mp-*cadA*	$Km^R$and Ap^R; pBV2mp derivative for *cadA* expression under control of the *mdh* promoter	This study
pAmy	Tet^R; pULMJ95 derivative for *Streptomyces griseus* IMRU 3570-derived *α-amylase* gene expression under control of the IPTG inducible promoter	(Seibold et al., 2006)
pTH1xpx-*amy*	Cm^R; pTH1xp derivative for *α-amylase* gene expression under control of the xylose inducible promoter	This study

Cm^R, chloramphenicol resistance; Km^R, kanamycin resistance; Ap^R, ampicillin resistance; Tet^R, tetracycline resistance.

Table S3. Primers used for the construction of plasmids. Bases underlined denote restriction recognition sites, bases in lower case denote overlapping regions, bases in bold denote mutations.

**Primer**	**Primer sequence 5'->3'**	**Purpose**
MCS1	GGCCACATGTTTAAAGCTTGAATTCCCGGGGATCCATATGGTACCCC	Insert for empty vector pTH1mp
MCS2	GGGGTACCATATGGATCCCCGGGAATTCAAGCTTTAAACATGTGGCC	Insert for empty vector pTH1mp
XPMF	ATACGGATCCGGTACCCCGCGGCCGCATCGATACATGTCCCGGGGAATTCACTGGCCGTCGTTT	Amplification of insert for pTH1xp
XPMR	GTTTCCCAAAACACCTATAC	Amplification of insert for pTH1xp
lacZF1	TGACATGTGCTAAAAAAACATGAAAAATTTTATTACG	Amplification of *lacZ* for pTH1mp-*lacZ*
lacZR1	AAGAATTCCTATTTTTCAATTACCTGC	Amplification of *lacZ* for pTH1mp-*lacZ*
MCSF	AGCGCTAGCATGCGGATCCGGTACCGAGCTCGAGATCTAGACTAGTA	Insert for promoter-less vector pTH1mcs-*lacZ*
MCSR	CATGTACTAGTCTAGATCTCGAGCTCGGTACCGGATCCGCATGCTAGC	Insert for promoter-less vector pTH1mcs-*lacZ*
lacZA	GGCCATGACGTCCTATTTTTCAATTACCTGCAAAATTTTC	Amplification of *lacZ* for pHCMC04-*lacZ*
lacZB	GGCCATGGATCCATGCTAAAAAAACATGAAAAATTTTATTAC	Amplification of *lacZ* for pHCMC04-*lacZ*
CUPF	AAGAGCTCTAGTCGAAGATTTTATGAAAG	Amplification of Cu^{2+}-inducible promoter for pTH1cup-*lacZ*

CUPR	ACACATGTGTTCACCACCTTTTCC	Amplification of Cu^{2+}-inducible promoter for pTH1cup-*lacZ*
MTLAF	TTTGCTAGCGGATAGTCTTGAATTTATTATTTGGAAGTATG	Amplification of the putative *mtlA*-promoter
MTLAR	TTTTCACATGTATTTCAACCCCTTTATCTACTATCAAC	Amplification of the putative *mtlA*-promoter
MDHPF	TTTGAATTCGTTCATTAAAGAGCAGCTG	Amplification of the *mdh*-promoter for pNW33Nmp
MDHPR	AAAGAGCTCGTCTCACATGTACTACCTCCTATTTATG	Amplification of the *mdh*-promoter for pNW33Nmp
KANF	AAAGATATCGGGCCAGTTTGTTGAAGATTAG	Amplification of the *kan* resistance gene for pNW33Nkan
KANR	AAAAAGCTTTGTACTGAGAGTGCACCATATGTC	Amplification of the *kan* resistance gene for pNW33Nkan
MDHP1	CATGGAGCTCATGGTACCTACCTCCTATTTATGTAATTG	Amplification of the *mdh*-promoter for pBV2mp
MDHP2	ATAGGATCCGCTGCAGTTCATTAAAGAG	Amplification of the *mdh*-promoter for pBV2mp
GFAF	tacataaataggaggtagtacatATGATTGAACAAGAGGTAC	Amplification of *gfpuv* for pTH1mp-*gfpuv*
GFPR	tggcgggtaccatatggatccTTATTTGTAGAGCTCATCCA	Amplification of *gfpuv* for pTH1mp-*gfpuv*
GBVF	cataaataggaggtaggtaccATGATTGAACAAGAGGTAC	Amplification of *gfpuv* for pBV2mp-*gfpuv*
GBVR	acgacggccagtgaattcgagctcTTATTTGTAGAGCTCATCCA	Amplification of *gfpuv* for pTH1mp-*gfpuv*
RoT781	CAGTGCCGACCAAAACCA	Amplification of pUB110 fragment with *ori*
RoT782	CAGCACAATTCCAAGAAAAA	Amplification of pUB110 fragment with *ori*
RoT783	tttttcttggaattgtgctgCTTAACGTGAGTTTTCGT	Amplification of pTH1mp fragment with *mp* and *ori*
RoT784	TAtggttttggtcggcactgCGCCATTCGCCATTCAGG	Amplification of pTH1mp fragment with *mp* and *ori*
MCHF	tacataaataggaggtagtacatATGGCGAGTAGCGAAGACGTTATC	Amplification of *mcherry* for pTH1mp-*mcherry*
MCHR	tggcgggtaccatatggatccTTAAGCACCGGTGGAGTGAC	Amplification of *mcherry* for pTH1mp-*mcherry*
PGF4	AGGAGGTAGTACATATGATTG	Amplification of pTH1mp-*gfpuv* fragment without *mp* promoter
PGR3	CTGCAGCCAAGCTTGGCGTAATCATGGTCATAGCTG	Amplification of pTH1mp-*gfpuv* fragment without *mp* promoter
MGAF	tacgccaagcttggctgcagCGTTTTGGAGGCGAGGCTGACCCATAA	Amplification of P_{mtlR} (m2p)
MGR2	aatcatatgtactacctcctAAGGAATTTAAAAAAAAACCAATC	Amplification of P_{mtlR} (m2p)
MMF1	CTATATTTTCCTTTCGTTG**A**A**A**TAAGGATATAAACGTT	SDM of -35 region of P_{mtlR} (m2p) promoter
MMR1	AACGTTTATATCCTTA**T**T**T**CAACGAAAGGAAAATATAG	SDM of -35 region of P_{mtlR} (m2p) promoter
MMF2	GGATATAAACGTTTTAT**A**ATAAATATGCAGGTATAATAT	SDM of -10 region of P_{mtlR} (m2p) promoter
MMR2	TATACCTGCATATTTAT**T**ATAAAACGTTTATATCCCTTA	SDM of -10 region of P_{mtlR} (m2p) promoter
MRF1	tacataaataggaggtagtacatATGTATATATCTGCAAGAGAAAG	Amplification of *mtlR*
MRR1	tggcgggtaccatatggatccTTATACACTCCTTAATTCTTTTAATTTTTC	Amplification of *mtlR*
MRF2	cagcctcgcctccaaaacgctgcagCAGATCCTTCTAATCCTTCTA	Amplification of *mtlR* with *mp* promoter
MFR2	ctatgaccatgattacgccaagcttTTATACACTCCTTAATTCTTTTAATT	Amplification of *mtlR* with *mp*

	TTTC	promoter
XPFW	GGATCCCCGGGTACCCCGCGGCCGCATCGA	Amplification of pTH1xp backbone
XPRW	GATTTAAGTGAACAAGTTTATC	Amplification of pTH1xp backbone
GXXF	gataaacttgttcacttaaatcAAAGGGGGAAATGACAAATGATTGAA CAAGAGGTAC	Amplification of *gfpuv* for pTH1xpx-*gfpuv*
GXRW	atgcggccgcggggtacccggggatccTTATTTGTAGAGCTCATCCA	Amplification of *gfpuv* for pTH1xpx-*gfpuv*
AX2F	gataaacttgttcacttaaatcAAAGGGGGAAATGACAAATGAAAAAAT TTTTTCTTACTTTCATCTTAATC	Amplification of *α-amylase* gene for pTH1xpx-*amy*
AX2R	atgcggccgcggggtacccggggatccTTAGGAGATTTTTTTTCTTCCTT TC	Amplification of *α-amylase* gene for pTH1xpx-*amy*

Table S4. Primers used for digital droplet PCR

Primer	Primer sequence 5'->3'	Target
CATF	CAGGAGTCCAAATACCAG AG	*cat*, chloramphenicol acetyltransferase (Cm^R)
CATR	CAATAGCGACGGAGAGTT AGG	*cat*, chloramphenicol acetyltransferase (Cm^R)
DNANF	GGACAGCAAGACAGATGT AG	*dnaN*, DNA polymerase III subunit beta (BMMGA3_00010)
DNANR	GGCTTTCGAACGTTGAG	*dnaN*, DNA polymerase III subunit beta (BMMGA3_00010)
pBM69F	CCCAGGAGCTTCTTATACG	replication protein pBM69 (BMMGA3_16760)
pBM69R	GGGTCGACTGAAAGGATA G	replication protein pBM69 (BMMGA3_16760)
pBM19F	GGCGGTTCTGCGGATTTA G	*repB*, replication protein pBM19 (BMMGA3_17180)
pBM19R	GTAGGGCCATTCCGTTGA C	*repB*, replication protein pBM19 (BMMGA3_17180)
pHCMC04F	GCCTCTAACTGTAACAGG AGTC	replication protein pHCMC04
pHCMC04R	GAAGGAATTGCGCTCTAT GC	replication protein pHCMC04
KNTF	GAACGTTTGGGCTTCTACC G	*knt*, kanamycin nucleotidyltransferase (Km^R)
KNTR	GATTGGCCGCTTACACAT GG	*knt*, kanamycin nucleotidyltransferase (Km^R)

Construction of plasmids used in the study

pTH1mp-*lacZ*: The *lacZ* gene was PCR-amplified from pNZlacZ using the primers lacZF1 and lacZR1, PCR product was digested by EcoRI and PciI and ligated into the corresponding sites of EcoRI and PciI-digested pTH1mp-*lysC*, generating pTH1mp-*lacZ*.

pTH1mcs-*lacZ*: An oligonucleotide insert was generated by dissolving the two oligos MCSF and MCSR in water to 100 µM and mixing them in a 1:1 ratio. 14µl of the mixture was combined with 2µl T4 DNA ligase buffer (New England Biolabs) and 0.5 µl polynucleotide kinase (PNK), and incubated at 37°C for 30 minutes and then at 65°C for 30 minutes to inactivate the PNK. 4µl of 1M NaCl was then added to the mix and the phosphorylated oligonucleotides annealed by incubation at 95°C for 10 min, followed by a gradual cool-down to room temperature. The annealed oligonucleotides were then ligated into PciI and SapI-digested pTH1mp-*lacZ*, generating the promoter-less pTH1mcs-*lacZ*.

pTH1xp-*lacZ*: The *lacZ* gene was PCR-amplified from pNZlacZ using the primers lacZA and lacZB, PCR product was digested by BamHI and AatII and inserted into the corresponding sites of pHCMC04, generating **pHCMC04-*lacZ***. This plasmid was then digested by NheI and PstI, and the fragment encoding the xylose-inducible promoter system and a part of *lacZ* was then ligated into the corresponding sites of pTH1mcs-*lacZ*, generating pTH1xp-*lacZ*.

pTH1cup-*lacZ*: A fragment encoding the cupper-inducible *atkY-atkB*-region was amplified from pRV613 using the primers CUPF and CUPR, PCR product was digested by SacI and PciI and ligated into the corresponding sites of pTH1mcs-*lacZ*, generating pTH1cup-*lacZ*.

pTH1mtlAp-*lacZ*: A fragment encoding the region upstream of *mtlA* was amplified from MGA3 genomic DNA using primers MTLAF and MTLAR, PCR product was digested by NheI and PciI and ligated into the corresponding sites of pTH1mcs-*lacZ*, generating pTH1mtlAp-*lacZ*.

pTH1xp was constructed by amplifying the fragment of pTH1mp with primers XPMF and XPMR, digesting PCR product and pTH1xp-*lacZ* with BamHI and NcoI, and ligating the resulting restriction products.

pTH1mp was constructed by cutting out the *lysC* gene of pTH1mp-*lysC* plasmid with PciI and KpnI to obtain linear pTH1mp. The insert was created by dissolving MSC1 and MSC2 primers in TE buffer to the final concentration of 100 µM, mixing them together in 1:1 ratio, incubating at 95°C for 10 min and leting them to gradually cool down at room temperature. The so created double-stranded oligomer was digested with PciI and Kpn and both fragments were ligated.

pNW33Nmp: The *mdh*-promoter was amplified from pTH1mp-*lysC* using the primers MDHPF and MDHPR, digested by EcoRI and SacI, and ligated into the corresponding sites of pNW33N, generating pNW33Nmp.

pBV2mp: The kanamycin resistance gene was amplified from pUCG18 using the primers KANF and KANR, PCR product was digested by EcoRV and HindIII and ligated into HincII-HindIII-digested pNW33N, generating **pNW33Nkan**. pNW33Nkan was digested by XbaI and HindIII and blunted using T4 DNA polymerase-mediated fill-in. The 954bp fragment encoding the kanamycin resistance gene was ligated into NheI and AflII-digested,T4 DNA polymerase blunted pHCMC04, generating **pHCMC04kan**, in which the chloramphenicol resistance gene has been replaced by the kanamycin resistance gene. Finally the *mdh*-promoter was PCR-amplified from pTH1mp-*lysC* using primers MDHP1 and MDHP2 and ligated into

BamHI and SacI-digested pHCMC04kan, thereby replacing the xylose-inducible promoter region, generating **pBV2mp**.

pUB110Smp was constructed by amplifying the fragment of pUB110 with primers RoT781 and Rot782 and the fragment of pTH1mp with primers RoT783 and RoT784, and joining the resulting PCR products by means of the isothermal DNA assembly method.

pTH1mp-*gfpuv* was constructed by amplifying *gfpuv* from pEPR1 with primers GFAF and GFPR, and joining the resulting PCR product with PciI and BamHI digested pTHmp by means of the isothermal DNA assembly method.

pBVmp-*gfpuv* was constructed by amplifying *gfpuv* from pEPR1 with primers GBVF and GBVR, and joining the resulting PCR product with KpnI and SacI digested pBVmp by means of the isothermal DNA assembly method.

pUB110Smp-*gfpuv* was constructed by amplifying *gfpuv* from pEPR1 with primers GFAF and GFPR, and joining the resulting PCR product with PciI and BamHI digested pUB110Smp by means of the isothermal DNA

pNW33Nmp-*gfpuv* was constructed by cutting *gfpuv* from pEPR1 of pTH1mp-*gfpuv* plasmid with BamHI and SpeI, and ligating the resulting restriction fragment with similarly treated pNW33Nmp.

pTH1mp-*mcherry* was constructed by amplifying *mcherry* with primers MCHF and MCHR, and joining the resulting PCR product with PciI and BamHI digested pTHmp by means of the isothermal DNA assembly method.

pTH1m2p-*gfpuv* was constructed by digesting the pTH1mp-*gfpuv* plasmid with Bsu36I and PstI and using this restriction product for amplification with PGF4 and PGR3, amplifying P_{mtlR} with MGAF and MGR2; and joining the resulting PCR products by means of the isothermal DNA assembly method.

pTH1m21p-*gfpuv* was constructed by site-directed mutagenesis (SDM) using primers MMF1and MMR1 of plasmid pTH1m2p-*gfpuv*.

pTH1m22p-*gfpuv* was constructed by site-directed mutagenesis (SDM) using primers MMF2and MMR2 of plasmid pTH1m21p-*gfpuv*.

pTH1m23p-*gfpuv* was constructed by site-directed mutagenesis (SDM) using primers MMF2and MMR2 of plasmid pTH1m2p-*gfpuv*.

pTH1Pm2p-*gfpuv* was constructed in two steps. First, pTH1mp-*mtlR* was was constructed by amplifying *mtlR* with primers MRF1and MRF1, and joining the resulting PCR product with PciI and BamHI digested pTHmp by means of the isothermal DNA assembly method. Next, the fragment containing the *mdh* promoter and *mtlR* was amplfied with MRF2 and MRR2, and the resulting PCR product was joined with PstI and HindIII digested pTH1m2p-*gfpuv* by means of the isothermal DNA assembly method.

pTH1xpx-*gfpuv* was constructed by amplifying *gfpuv* from pEPR1 with GXXF and GXRW, amplifying pTH1xp backbone with XPFW and XPRW, and joining the resulting PCR products by means of the isothermal DNA assembly method.

pBV2mp-*cadA*: pTH1mp-*cadA* was digested by FspI and SpeI and the 2.4kb fragment encoding *cadA* was ligated into Eco53kI and SpeI-digested pBV2mp, generating pBV2mp-*cadA*.

pTH1xpx-*amy* was constructed by amplifying *α-amylase* gene from pAmy plasmid with AX2F and AX2R, amplifying pTH1xp backbone with XPFW and XPRW, and joining the resulting PCR products by means of the isothermal DNA assembly method.

References

Brautaset, T., Jakobsen, Ø. M., Degnes, K. F., Netzer, R., Nærdal, I., Krog, A., et al. (2010). *Bacillus methanolicus* pyruvate carboxylase and homoserine dehydrogenase I and II and their roles for L-lysine production from methanol at 50°C. *Appl. Microbiol. Biotechnol.* 87, 951–964. doi:10.1007/s00253-010-2559-6

Cleto, S., Jensen, J. K., Wendisch, V. F., Lu, T. K. (2016). *Corynebacterium glutamicum* metabolic engineering with CRISPR interference (CRISPRi). *ACS Synth. Biol.* [Epub ahead of print]. doi:10.1021/acssynbio.5b00216

Crutz-Le Coq, A. M., Zagorec, M. (2008). Vectors for lactobacilli and other Gram-positive bacteria based on the minimal replicon of pRV500 from *Lactobacillus sakei*. *Plasmid* 60, 212–220. doi:10.1016/j.plasmid.2008.08.002

Gryczan, T. J., Contente, S., Dubnau, D. (1978). Characterization of *Staphylococcus aureus* plasmids introduced by transformation into *Bacillus subtilis*. *J. Bacteriol.* 134, 318–29.

Haima, P., Bron, S., Venema, G. (1987). The effect of restriction on shotgun cloning and plasmid stability in *Bacillus subtilis* Marburg. *Mol. Gen. Genet.* 209, 335–342. doi:10.1007/BF00329663

Knoppova, M., Phensaijai, M., Vesely, M., Zemanova, M., Nesvera, J., Patek, M. (2007). Plasmid vectors for testing in vivo promoter activities in *Corynebacterium glutamicum* and *Rhodococcus erythropolis*. *Curr. Microbiol.* 55, 234–239. doi:10.1007/s00284-007-0106-1

Kovács, A. T., van Hartskamp, M., Kuipers, O. P., van Kranenburg, R. (2010). Genetic tool development for a new host for biotechnology, the thermotolerant bacterium *Bacillus coagulans*. *Appl. Env. Microbiol.* 76, 4085–4088. doi:10.1128/AEM.03060-09

Nærdal, I., Pfeifenschneider, J., Brautaset, T., Wendisch, V. F. (2015). Methanol-based cadaverine production by genetically engineered *Bacillus methanolicus* strains. *Microb. Biotechnol.* 8, 342–350. doi:10.1111/1751-7915.12257

Nguyen, H. D., Nguyen, Q. A., Ferreira, R. C., Ferreira, L. C. S., Tran, L. T., Schumann, W. (2005). Construction of plasmid-based expression vectors for *Bacillus subtilis* exhibiting full structural stability. *Plasmid* 54, 241–248. doi:10.1016/j.plasmid.2005.05.001

Rhee, M. S., Kim, J., Qian, Y., Ingram, L., Shanmugam, K. (2007). Development of plasmid vector and electroporation condition for gene transfer in sporogenic lactic acid bacterium, *Bacillus coagulans*. *Plasmid* 58, 13–22. doi:10.1016/j.plasmid.2006.11.006

Seibold, G., Auchter, M., Berens, S., Kalinowski, J., Eikmanns, B. J. (2006). Utilization of soluble starch by a recombinant *Corynebacterium glutamicum* strain: growth and lysine production. *J. Biotechnol.* 124, 381–391. doi:10.1016/j.jbiotec.2005.12.027

Taylor, M. P., Esteban, C. D., Leak, D. J., 2008. Development of a versatile shuttle vector for gene expression in *Geobacillus* spp. *Plasmid* 60, 45–52. doi:10.1016/j.plasmid.2008.04.001
